# Loss of endothelial cell-specific molecule 1 promotes the tumorigenicity and metastasis of prostate cancer cells through regulation of the TIMP-1/MMP-9 expression

**DOI:** 10.18632/oncotarget.14684

**Published:** 2017-01-17

**Authors:** Chien-Min Chen, Chu-Liang Lin, Hui-Ling Chiou, Shu-Ching Hsieh, Chia-Liang Lin, Chun-Wen Cheng, Chia-Hung Hung, Jen-Pi Tsai, Yi-Hsien Hsieh

**Affiliations:** ^1^ Division of Neurosurgery, Department of Surgery, Changhua Christian Hospital, Changhua, Taiwan; ^2^ School of Medicine, Kaohsiung Medical University, Kaohsiung, Taiwan; ^3^ Institute of Biochemistry, Microbiology and Immunology, Chung Shan Medical University, Taichung, Taiwan; ^4^ School of Medical Laboratory and Biotechnology, Chung Shan Medical University, Taichung, Taiwan; ^5^ School of Medicine, Tzu Chi University, Hualien, Taiwan; ^6^ Division of Nephrology, Department of Internal Medicine, Dalin Tzu Chi Hospital, Buddhist Tzu Chi Medical Foundation, Chiayi, Taiwan; ^7^ Department of Biochemistry, School of Medicine, Chung Shan Medical University, Taichung, Taiwan; ^8^ Clinical laboratory, Chung Shan Medical University Hospital, Taichung, Taiwan

**Keywords:** prostate cancer cells, endothelial cell specific molecule 1, tumorigenicity, metastasis, epithelial-mesenchymal transition

## Abstract

The Endothelial cell specific molecule-1 (ESM1) protein has been involved in proliferation and metastatic progression in multiple tumors. However, there are no studies regarding the mechanism of ESM1 in prostate cancer. We found that ESM1 knockdown in prostate cancer cells resulted in increased cell proliferation and colony formation ability response evidenced by decreased expression of p21 and increased expression of cyclin D1 in prostate cancer cells. Moreover, we revealed that knockdown ESM1 also induced the epithelial-mesenchymal transition (EMT), motility and invasiveness in accordance with the upregulated the MMP-9 expression, while downregulated the TIMP-1 expression. Recombinant human (Rh) TIMP-1 significantly attenuated ESM1-mediated cell migration and invasion. Additionally, ESM1 knockdown increased *in vivo* tumorigenicity and metastasis of prostate cancer cells. These findings provide the first evidence that the imbalance of MMP-9/TIMP-1, is one of the regulation mechanisms by which ESM1 promotes tumorigenicity and metastasis of prostate cancer cells.

## INTRODUCTION

Prostate cancer is the common type of cancer and is a second leading cause of cancer-related death in men worldwide [1]. Risk factors for the development of prostate cancer including longer life expectancy, diets high in red meat, inherited prostate-cancer susceptibility genes and chronic inflammation [2, 3]. Prognosis of prostate cancer depended on tumor stage, grade, pre-therapy prostate specific antigen level and Gleason score, which indicated that patients with local or regional prostate cancers had a much better survival rate than those with distant metastasis [4]. In addition to the well-known modalities of treatment including operation, radiotherapy or hormone therapy, patients who develop androgen-independent prostate cancer after radiation therapy or hormone ablation therapy and those who had metastatic disease at the time of diagnosis are much less to be cured and with dismal survival [5, 6]. The mechanisms of biological capacities developed during the development of prostate cancer, including proliferative signaling, evading growth factors, resisting cell death, enhancing replicative immortality, inducing angiogenesis, invasion and metastasis, were still remained to be elucidated [2].

The malignant cells must develop a complex of molecular changes that regulated cell morphology and function, the so-called epithelial-mesenchymal transition (EMT), to destroy intercellular relationship and cell-matrix adhesive characteristics, break down the extracellular matrix (ECM), and breach the basement membrane by the modulation of matrix metalloproteinases (MMP) to be motile and invasive, [7–9]. EMT had been known to involved in cancer progression, which was often marked by decreased expression of E-cadherin (epithelial marker) and increased expression of N-cadherin and Vimentin (mesenchymal markers), and the alternations of these proteins resulted in the impairment of cell–cell adhesion and the spreading cancer cells [10]. Zhao et al. found the expression of vimentin and E-cadherin was associated with the motility and invasiveness of prostate cancer cells [11]. In addition, MMPs are a family of zinc-dependent endopeptidases with broad substrate specificities for a variety of ECM or basement membrane components, such as collagen, laminin and fibronectin [12]. Recently, Lichtinghagen et al. had reported that expression of MMP-9 and the ratio of MMP-2 and MMP-9 to the tissue inhibitor of metalloproteinase 1 to be higher in cancerous prostate tissues than in normal prostate tissues [13].

Endothelial cell specific molecule-1 (ESM1), which was a soluble proteoglycon of 50 kDa and constituted of a mature polypeptide of 165 amino acid, was originally cloned from a human endothelial cDNA library by Lassalle and colleagues in 1996 [14]. The expression of ESM1 in normal human tissues was selectively in neogenetic or active tissues, such as glandular or tubular epithelium or a variety of organs, lymph node and capillary endothelial cells [15]. Moreover, the levels of mRNA of ESM1 were found to be highly regulated by inflammatory cytokines, such as tumor necrosis factor α and interleukin 1, and be linked with tumor growth and angiogenic process during tumor progression [14, 16, 17]. After the first observation of properties of promoting tumor growth of ESM1 from engineered HEK293 cells in a mouse model of human tumor xenograft [17], ESM1 was later found to be correlated with lung, renal and breast cancers [18–21]. In addition, microvessel density denoted by ESM1 was correlated with microscopic venous invasion and vascular endothelial growth factor expression in hepatocellular carcinoma patients [22], and the blood levels of circulating ESM1 were found to markedly increase in the sera of advanced lung cancer patients [17]. Moreover, ESM1 had been suggested to play a role on tumor metastasis, migration and vascular invasion in human hepatocellular carcinoma, gastric and colorectal cancers by regulating the expression of MMPs [23–25]. It had been reported that ESM1 was associated with several types of malignancies, but the roles of ESM1 on prostate cancer remained unclear. So, we conducted this study to investigate the prostate cancer cells *in vitro* and *in vivo* to explore the function and underlying molecular mechanisms of ESM1 on human prostate cancer cells.

## RESULTS

### Knockdown of ESM1 increased the prostate cancer cells proliferation

**Figure 1:**
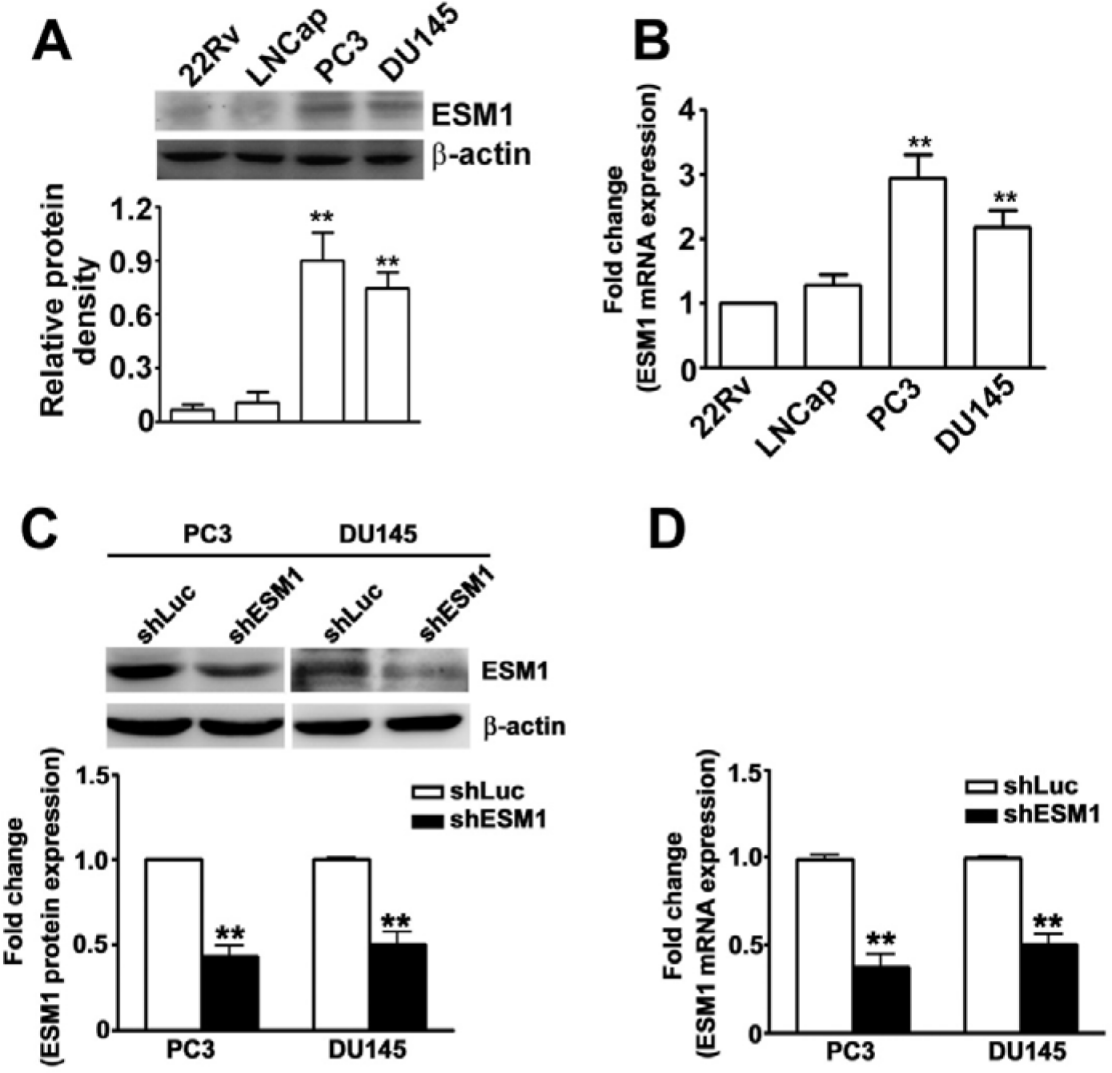
Expression of ESM1 in prostate cancer cells and knockdown ESM1 on the expression of ESM1 of PC3 and DU145 cells ****(A)**** Total lysate from PC3, DU145, and 22Rv1, LNCap cells were isolated and analyzed by western blotting **(B)** Total RNAs were isolated and then qRT-PCR assay was applied to detect ESM1 mRNA expression. **(C)** PC3 and DU145 cells were infected with shLuc or shESM1 and then purpomylin (2 or 10 mg/ml) for 5 days. Then, total lysates were isolated and analyzed by western blotting. **(D)** qRT-PCR assay was applied to detect ESM1. β-actin was used as internal control for protein equal loading. Values are expressed as the mean ± SE of three independent experiments. **p < 0.01.

**Figure 2:**
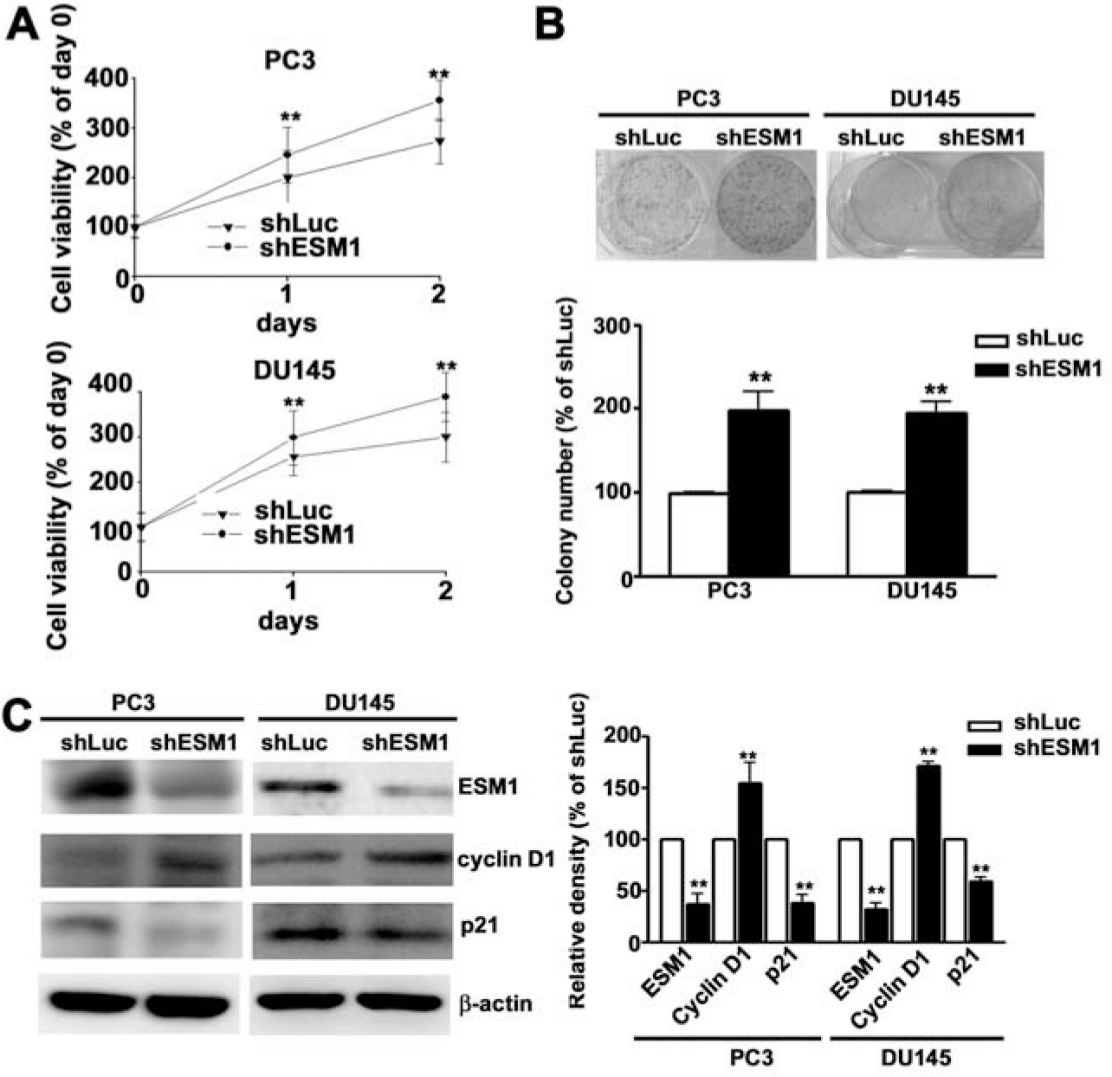
Knockdown of ESM1 on the proliferation of PC3 and DU145 cell lines **(A)** The shLuc or shESM1-PC3 and -DU145 cells on the cell viability were evaluated using a MTT assay after 1 and 2 days. **(B)** The clonogenic ability of shLuc or shESM1-PC3 and shESM1-DU145 cells were incubated for 14 days and total colony numbers were calculated. **(C)** Western blots analysis on ESM1, cyclin D1 and p21 expression in shLuc or shESM1-PC3 and shESM1-DU145 cells. Quantification of migrated cells was shown as a histogram chart. Data are presented as the mean ± SE of at least three independent experiments. β-actin was used as internal control for protein equal loading. **p < 0.01, compared with shLuc cells.

Western blot analysis and qRT-PCR detected ESM1 protein in four of the prostate cancer cell lines (PC3, DU145, 22Rv1 and LNCap) examined. Western blotting and qRT-PCR results confirmed the upregulation of ESM1 protein and mRNA expression in the PC3 and DU145 cells (Figure 1A, 1B). We chose the PC3 and DU145 cells for the subsequent study. To study biological consequences of ESM1 upregulation in prostate cancer cells, our data revealed that stably expressing ESM1 shRNA in PC3 and DU145 cells, ESM1 protein and mRNA expression were significantly reduced compared to the shLuc cells by western blotting and qRT-PCR analysis (Figure 1C and 1D). Cell proliferation is necessary for tumor cell growth, PC3 and DU-145 cells exhibiting stable ESM1 knockdown showed enhanced cell proliferation by MTT assay (Figure 2A) and increased colony formation ability by foci formation assays (Figure 2B). To explore the mechanism leading to the increased proliferation of ESM1 knockdown cells by western blotting assay. We found that ESM1 knockdown were significantly decreased expression of p21, whereas the expression of cyclin D1 was significantly increased in shESM1-PC3 and shESM1-DU145 cells compared to shLuc cells (Figure 2C). Furthermore, the proliferative capacity in ESM1 overexpressing shESM1-DU145 cells was significantly lower than in shESM1-DU145 cells (Supplementary Figure 1A). Identically, overexpression of ESM1 in shESM1-DU145 cells resulted in increased p21 levels and decreased cyclin D1 levels (Supplementary Figure 1B). These results suggest that ESM1 plays an important role in regulating prostate cancer cells proliferation.

### ESM1 knockdown promotes cell migration and invasion, and alters the expression of MMP-9/TIMP-1

**Figure 3:**
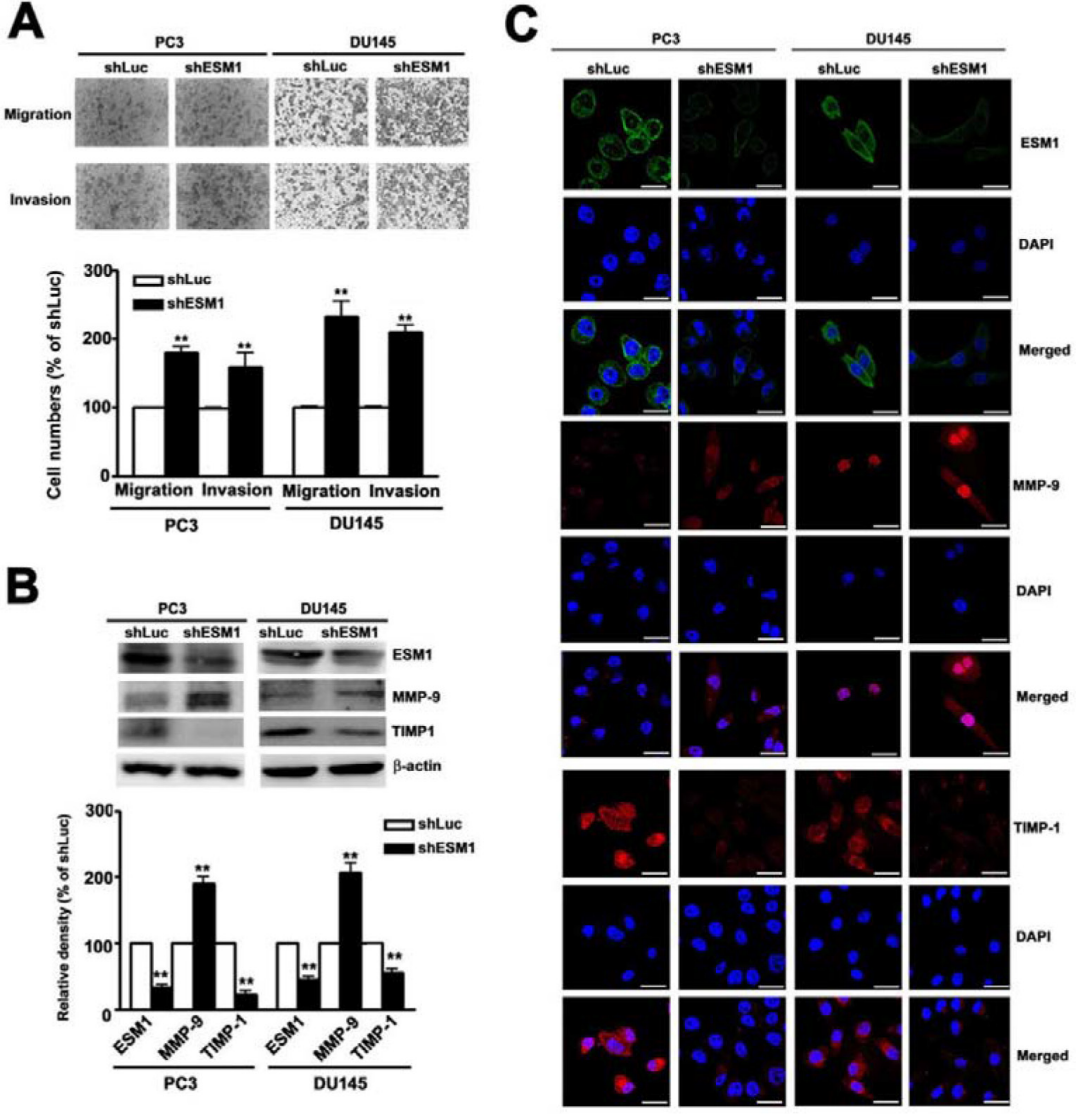
Knockdown of ESM1 on the migration and invasion of PC3 and DU145 cell lines **(A)** The abilities of migration and invasion of shLuc or shESM1-PC3 and shESM1-DU145 cells were determined by using a migration assay and Matrigel-invasion assay. Cells in the lower surface of the Borden chamber were stained and photographed under a light microscope at ×400 magnification. Quantification of migrated cells was shown as a histogram chart. **(B)** Western blots analysis on ESM1, MMP-9 and TIMP-1 expression in shLuc or shESM1-PC3 and shESM1-DU145 cells. β-actin was used as internal control for protein equal loading. **(C)** The immunofluorescence staining of ESM1, MMP-9, and TIMP-1 expression. DAPI staining of nucleus in each cell line. Data are presented as the mean ± SE of at least three independent experiments. **p < 0.01, compared with shLuc cells. Scale bars = 100 mm.

The human prostate cancer cell line PC3 and DU-145 were further validating the effect of ESM1 on the migratory and invasive behavior of prostate cancer cells. The *in vitro* migration and invasion assay results showed that knockdown ESM1 was significantly increased the migration and invasion in shESM1-PC3 and shESM1-DU145 cells compared to shLuc cells (Figure 3A). The balance between MMP-9 and TIMP-1 are reported to play a critical role of migration and invasion by stimulating degradation of the ECM in prostate cancer cell and is associated with enhanced tumor metastatic potential [7]. Knockdown ESM1 expression leads to a significant increase the MMP-9 expression and decrease the TIMP-1 expression in shLuc-PC3 and shLuc-DU145 cells compared to shLuc cells (Figure 3B), Similar results were obtained in immunofluorescence assay (Figure 3C). Moreover, overexpression of ESM1 inhibited cell migration and invasion of shLuc-DU145 cells, compared to shLuc-DU145 cells (Supplementary Figure 2A). We also found that significantly reduced MMP-9 levels and significantly increased TIMP-1 levels (Supplementary Figure 2B) by western blotting. These results indicated that knockdown ESM1 enhanced the abilities of migration and invasion of prostate cancer cells through regulation of the TIMP-1/MMP-9 expression.

### ESM1 knockdown promotes epithelial-mesenchymal transition of prostate cancer cells

**Figure 4:**
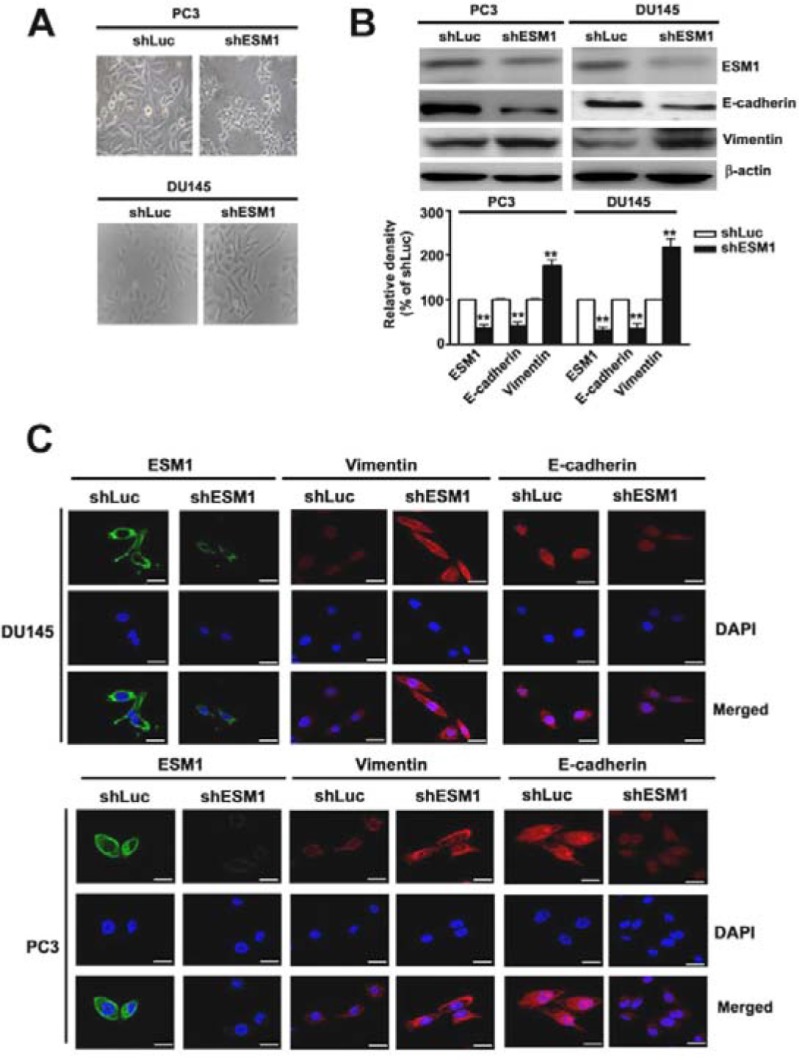
Knockdown of ESM1 on the epithelial-mesenchymal transition of PC3 and DU145 cell lines **(A)** The morphological appearance of shLuc or shESM1-PC3 and shESM1-DU145 cells and **(B)** then the cell lysates were subjected to western blots analysis on ESM1, vimentin and E-cadherin expression. **(C)** The immunofluorescence staining of ESM1, vimentin and E-cadherin expression, and cell nuclei were counterstained with DAPI reagent. Data are presented as the mean ± SE of at least three independent experiments. **p < 0.01, compared with shLuc cells. Scale bars = 100 mm.

Another characteristic of tumor metastasis is that the cells lose one phenotype and acquired a new one, the EMT. During the phenotype conversion, there is an induction of mesenchymal markers, such as Vimentin, and disappearance of epithelial markers of epithelial cells, like E-cadherin, which is essential for the structural integrity of epithelium [26]. Therefore, the morphogenic appearance of shESM1-PC3 and shESM1-DU145 cells were observed and revealed disordered alignment cpmpared with shLuc cells (Figure 4A). We also found that significantly increased expression of vimentin, and decreased expression of ESM1 and E-cadherin in shESM1-PC3 and shESM1-DU145 cells, compared with shLuc cells (Figure 4B). These observations were corroborated by the results obtained with the immunofluorescence assay (Figure 4C). These results indicated that knockdown of ESM1 could promote the trans-differentiation of epithelial cells to mesenchymal cells of prostate cancer cells.

### Overexpression of TIMP-1 attenuated ESM1 inhibiting induces migration and invasion of PC3 cells

**Figure 5:**
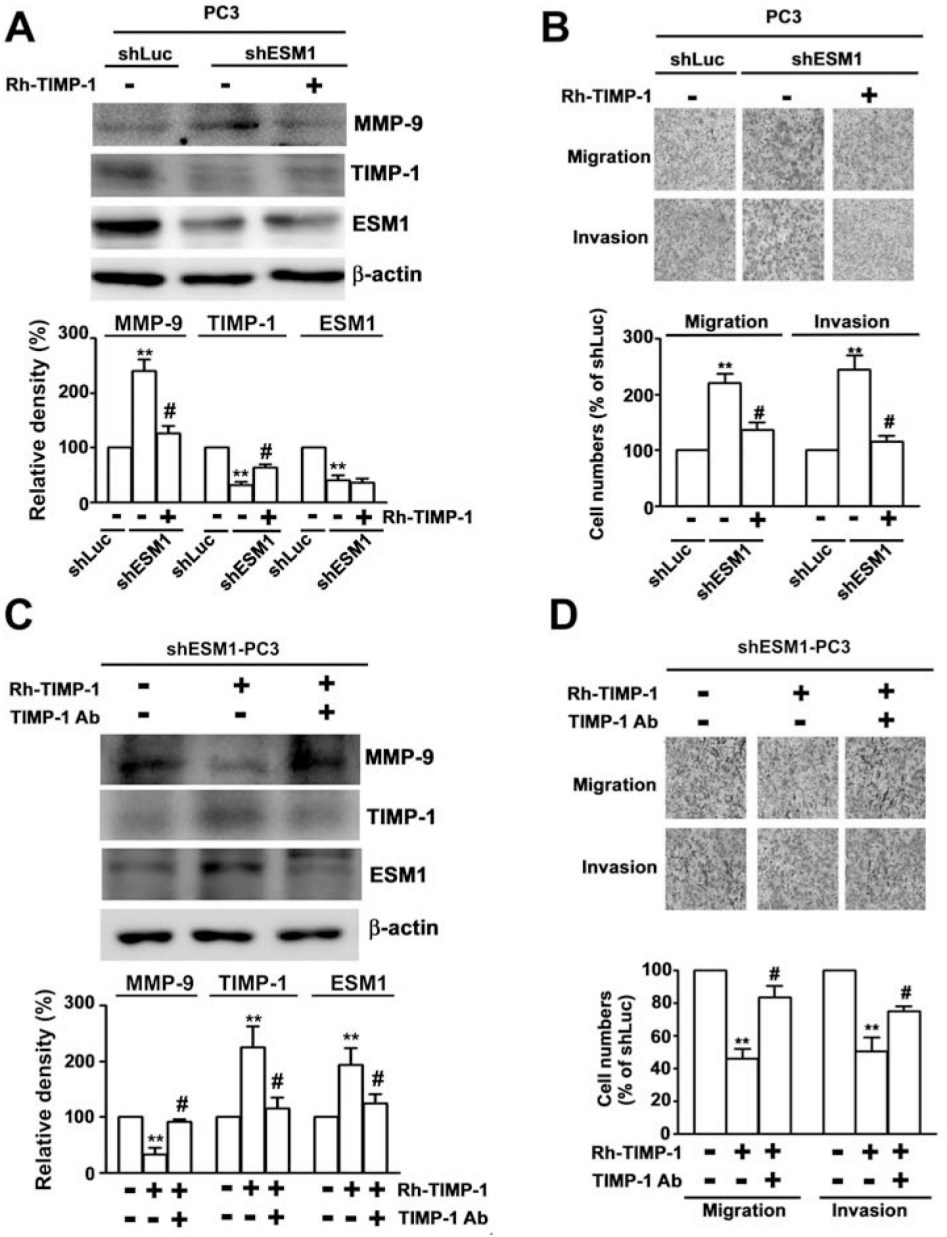
Rh-TIMP-1 attenuates knockdown ESM1 induces migration and invasion in PC3 cells The shLuc and shESM1-PC3 cells were treated with 100 ng/mL RhTIMP-1 or cotreated with 100 ng/ml and 1mg TIMP-1 antibody for 24 h. **(A, C)** Protein expression of ESM1, MMP-9 and TIMP-1, as measured with western blotting. **(B, D)** The abilities of migration and invasion of cells were determined by using migration and invasion assay. Quantification of migrated cells was shown as a histogram chart. **p < 0.01, compared with shLuc cells. #p < 0.01, compared with Rh-TIMP-1 treated cells.

To further understand the function of TIMP-1 in prostate cancer, we studied the effect of recombinant-TIMP-1 (Rh-TIMP-1) on cell migration and invasion of prostate cancer cells. Western blot analysis showed that shESM1-PC3 cells treated with Rh-TIMP-1 (100 ng/ml) had increased TIMP-1 expression and decreased MMP-9 expression (Figure 5A). The migration and invasion assay analysis showed that shESM1-PC3 cell migration and invasion was significantly decreased by Rh-TIMP-1 expression compared to shESM1-PC3 cells (Figure 5B). By contrast, anti-TIMP-1 antibody with Rh-TIMP-1 (Figure 5C). To evaluate the biologic role of TIMP-1 in the PC3 cell migration and invasion, PC3 cells were incubated with anti-TIMP-1 antibody in *in vitro* migration and invasion assay prior to determination of cell migrates and invasiveness. The result showed that cells treated with Rh-TIMP-1 (100 ng/ml) had decreased the migration and invasion of PC3 cells, in contract, PC3 cells co-treated with anti-TIMP-1 antibody and Rh-TIMP-1 were significantly increased migration and invasion, compared with Rh-TIMP-1 only treatment (Figure 5D). These results suggest that TIMP-1 overexpression may be promote ESM1 expression via regulation of cell migration and invasion in human prostate cancer cells.

### ESM1 knockdown increased the tumorigenesis and metastasis of prostate xenograft tumours in nude mice

**Figure 6:**
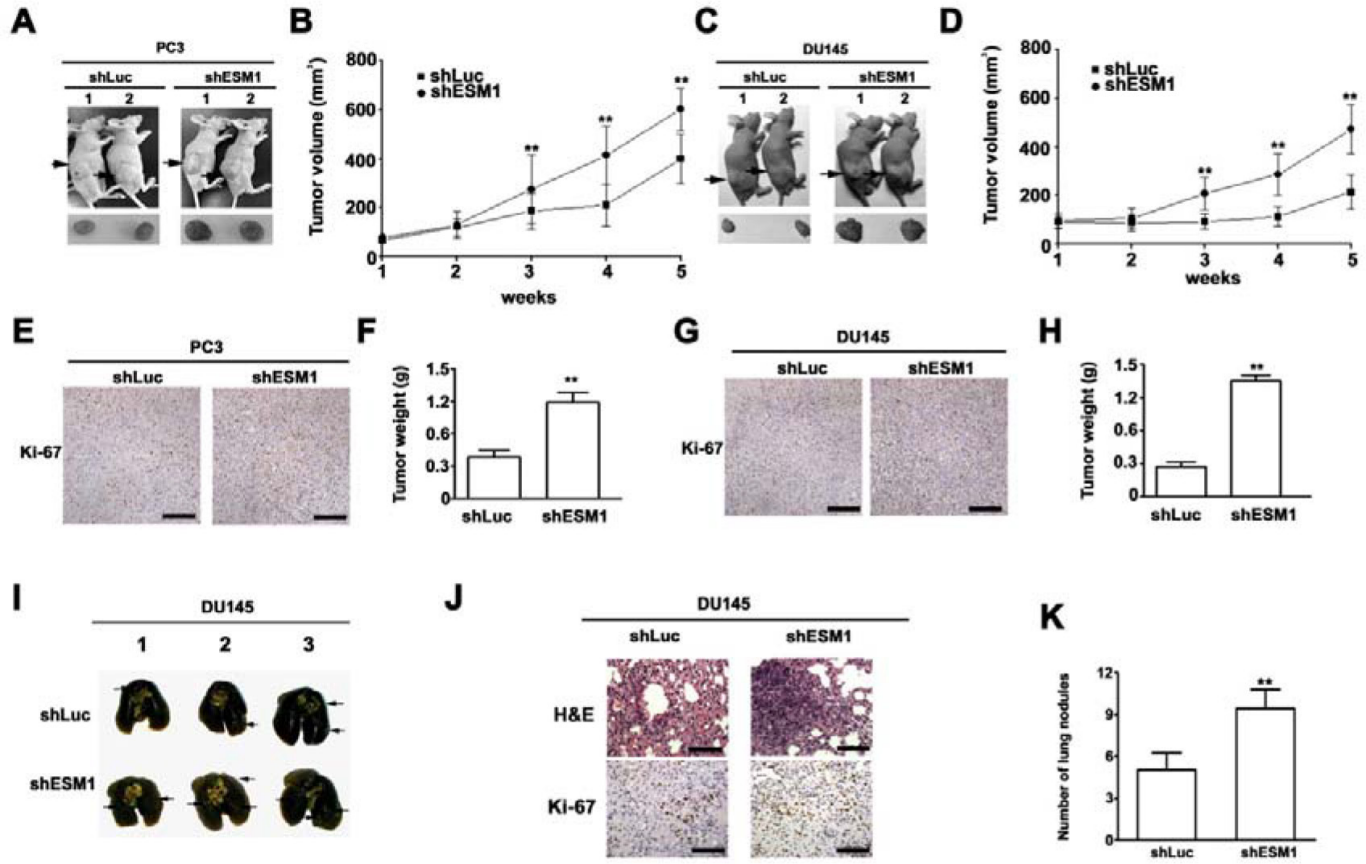
Knockdown of ESM1 enhances tumor growth and metastasis in prostate cancer cells in vivo (**A** and **C**) Nude mice being inoculated with shLuc- or shESM1-PC3 and shESM1-DU145 cells were observed until study termination. (**B** and **D**). The growth of tumors was monitored in terms of tumor volume every week. At the termination of the experiment, the tumors were excised after the mice were sacrificed, and the tissue samples of excised xenograft tumors were examined with immunohistochemical stain with anti-Ki-67 antibody (×100). (**E** and **G**) and the weights of the tumors (**F** and **H**) were determined. Scale bars = 100 mm. (**I**) Mouse lungs showed the metastatic ability of shLuc group and shESM1 group. (**J**) Then the metastatic nodules were calculated and (**K**) the lungs were fixed in 4% paraformaldehyde and sectioned for H&E staining and immunohistochemical stain with anti-Ki-67 antibody. **p < 0.01, compared with shLuc group. Scale bars = 50 mm.

Because inhibition of ESM1 could enhance the abilities of proliferation, migration and invasion of prostate cancer cells *in vitro*, it was necessary to examine whether this effect could enhance the progression of prostate cancer cells *in vivo*. To test the effects of shESM1 on the proliferation of prostate cancer cells, shLuc-/shESM1-PC3 and shLuc-/shESM1-DU145 cells implanted into nude mice, tumor growth was substantially elevated with shESM1-PC3 as compared with shLuc-PC3 cells (Figure 6A, 6B). These results are similar to shESM1-DU145 cells (Figure 6C, 6D). The expression of Ki-67 in the shESM1-PC3 group and shESM1-DU145 group were significantly higher than that in the shLuc-PC3 group by immunohistochemical staining assays (Figure 6E, 6G). On the 35 days after tumor appearance, the average tumor weight in the shESM1-PC3 group and shESM1-DU145 group were significantly higher than those in the shLuc-PC3 group and shLuc-DU145 groups (Figure 6F, 6H).

The impact of ESM1 on prostate metastasis was performed in a model of prostate metastatic spreading by injected intravenously in the tail vein of shLuc-DU145 and shESM1-DU145 in the nude mice. After 8 weeks, metastatic nodules at the lung surfaces were resected, photographed and counted. The numbers of metastatic nodules were counted grossly and those mice inoculated with shESM1-DU145 group grew more nodules than those inoculated with shLuc group (Figure 6I, 6K). Afterwards, the excised metastatic nodules were examined by H&E stain and immunohistochemical stain with anti-Ki-67 antibody. Compared to mice inoculated with shLuc group, these tumor samples from those inoculated with shESM1-DU145 cells showed distinctly more cells displaying mitotic features and increase of Ki-67 positive cells by H&E stain and immunohistochemical stain, respectively (Figure 6J). Taken together, these data indicated that ESM1 knockdown promoted prostate cell growth and metastasis in nude mice.

## DISCUSSION

Prostate cancer is the most frequently diagnosed and one of the leading causes of cancer-related death in men [1]. While localized prostate cancer can be treated by surgical resection or through radiation therapy, the prognosis of patients with advanced or metastatic diseases remained suboptimal, as these tumors respond poorly to standard cytotoxic regimens [5, 6]. Although androgen ablation remained the primary course of treatment for all patients with metastatic disease through induing cell death or cell cycle arrest of prostate cancer cells, these therapies became ineffective wherein androgen signaling being inappropriately expressed [2]. In addition to the well-known biological capacities acquire during the progress of cancer, inflammation had been elucidated to contribute to foster the neoplastic progression by supplying bioactive molecules to the tumor micro-environment, which included growth factors, survival factors, pro-angiogenic factors, or extracellular matrix modifying enzymes that lead to angiogenesis, invasion and metastasis [2, 27]. Endothelial cell specific molecule-1 was a soluble proteoglycon and was recently been known to associate with several mechanisms that involved in the progression and prognosis of multiple malignancies [20, 21]. Studies using microarray and specific antibodies against ESM1 had revealed ESM1 to be a candidate biomarker in various cancers, including lung, breast, colon and kidney cancers [18–21]. Moreover, over-expression of ESM1 had been reported to be correlated with vascular endothelial growth factor, which could therefore represented as a biomarker of endothelial cell activation in response to pro-angiogenic signals from hepatocellular carcinoma [22]. We further confirmed that ESM1 knockdown on the growth and metastasis of prostate cancer cells *in vitro* and *in vivo*, suggesting the tumor suppressor role of ESM1 in prostate cancer.

In this study, we first suggested inhibited ESM1 expression promoted prostate cell proliferation. We then infected a lentivirus system expressing ESM1-specific shRNA, and suggested that knockdown of ESM1 in PC3/DU145 cells significantly increased cell proliferation as well as colony-forming ability. Cell proliferation was strictly controlled by cell cycle checkpoints, and the regulation of cell cycle in human prostate cancer had shown to have multiple abnormal signaling pathways with aberrant production of several cell-cycle related proteins [28]. Previous study demonstrated that ESM1 could had a role on regulating cell cycle. After treating with ESM1 siRNA in SK-Hep1 hapatocellular carcinoma cells, the cell cycle was arrested at the G1/S phase with down-expression of cyclin D1 and CKD4, but up-expression of PTEN, which had tumor suppressing function [25]. Similarly, colorectal cancer cells transfected with ESM1 siRNA resulted in cell cycle arrested at G1 phase by inducing PTEN and inhibition of cyclin D1 [24]. In our study, knockdown of ESM1 showed up-regulation of cyclin D1 and down-regulation of p21 with the results of increased proliferation of prostate cancer cells. Being different from these previous studies, our study indicated that inhibiting the expression of ESM1 could enhance cell growth by modulating cell-cycle related proteins of prostate cancer cells.

By the aberrant regulation of the MMPs to destroy the intercellular relationship, lyse the ECM and breach the basement membrane, malignant cells could have the abilities of migration and invasion to become distant metastasis [8, 9]. Lichtinghagen et al. had reported that expression of MMP-9 and the ratio of MMP-2 and MMP-9 to the TIMP-1 to be significantly higher in prostate cancer tissues than in normal prostate tissues [13]. Similarly, Mahta et al. reported high expression of mitogen/extracellular-signal-regulated kinase 5 in human prostate cancers and correlated with the presence of bony metastasis, and profound expression of MMP-9 was found through enhancing the activities of activator protein-1 after transfection experiments [29]. Moreover, Takaha et al. transfected prostate cancer cells with high mobility group protein and revealed enhanced ability of invasion by overexpression of MMP-2 proform [30]. Furthermore, expression of ESM1 had been reported to be correlated with metastasis, migration and vascular invasion in human gastric and colorectal cancer [23, 24]. It had been reported that knockdown of ESM1 gene expression by transfecting SK-Hep1 hepatocellular carcinoma cells with ESM1 siRNA showed 1.6-fold and 2.2-fold decreased abilities of migration and invasion [25]. Previous study reported ESM1 as a potential serum marker for early detection of colorectal cancer and later demonstrated that ESM1 could play a role in inhibiting cyclin D1 and cell migration and invasion through modulating G1 phase cell cycle by induction of PTEN and in modulating cell proliferation through the Akt-dependent activation of NF-κB pathway [24]. Some studies showed that vimentin transfected cells showed more vimentin expression and less E-cadherin expression and furthermore led to a significant increase in prostate cancer cells motility and invasive activities resulting in distant pulmonary metastasis [11, 31]. We speculated that knocking down of the expression of ESM1 could enhance the phenotypic changes, increase the abilities of migration and invasion by increasing over-expression of MMP-9 and down-expression of TIMP-1, and aggressiveness of distant metastasis of prostate cancer cells, which indicated a potential role of ESM1 in regulating the progress of prostate cancer.

Taken together, ESM1 could play a tumor suppressing role against the growth, proliferation, and metastasis of prostate cancer cells in this study, which was contrary to previous studies [23–25]. Therefore, we considered that there seem to be another mechanism modulated by ESM1 against the progression of prostate cancer. Zuo et al. had found that ESM1 expression was down-regulated in cancerous tissues than in normal tissues, and scarcely expressed in poor-differentiated colorectal carcinoma [32]. Recently, Yassine et al. conducted a study regarding the role of ESM1 on tumor progression in mice and found that endogenous over-expression or systemic administration of ESM1 could retard the growth in HT-29 tumor cells. The authors considered that since human fully glycanated ESM1 could promote tumor growth, there was one interesting hypothesis regarding the balance between glycanated and non-glycanated ESM1, which could induce tumor growth or retardation [33]. In addition, they suggested a chemokinetic property of human ESM1 to inhibit the interaction of leukocyte integrin LFA-1 and adhesion molecule ICAM-1 by the participation of CD 122^+^ leukocytes [33, 34]. This hypothesis was consistent with the molecular pathogenesis of prostate cancer supposed by Nelson et al. that prostate infection or inflammation initially caused a prostate-cancer-precursor lesion, the proliferative inflammatory atrophy, and then initiated prostatic carcinogenesis [2].

The biological role of TIMP-1 in reducing tumor invasiveness is well established [35]. Similar results suggested that knockdown of Cul1 induces cell cycle arrest at the G0/G1 phase. Furthermore, knockdown of Cul1 suppresses renal cancer cell migration and invasion abilities by up-regulating the expression of TIMP-1and inhibit the expression of MMP-9 [36]. Indeed, other studies have found that silencing PRDX3 promoted invasive properties of HepG2 cells via TIMP-1 down-regulation and the increased ECM degradation [37]. In our study suggest a causal link between ESM1 knockdown, TIMP-1 inactivation, and increased invasiveness of PC3 and DU145 cells.

To the best of our knowledge, this is the first study to demonstrate the role and possible mechanism of ESM1 on the progression of human prostate cancer cells. We demonstrated down-expression of ESM1 could enhance the viability, abilities of migration and invasion, and aggressiveness of human prostate cancer cells *in vitro* and *in vivo* by modulating cell-cycle related proteins, by controlling the expression of MMP-9 and TIMP-1, and by enhancing the phenotypic changes to cell proliferation. In conclusion, although the precise mechanism by which ESM1 exerted its effects on the progression of prostate cancer remained to be determined, our present findings suggested that ESM1 could modulate the progression of prostate cancer by methods as tumor suppression in this study.

## MATERIALS AND METHODS

### Antibodies and reagents

Antibody against ESM1 (ab103590) was purchased from Abcam (Cambridge. MA). Antibodies against p21 (sc-397; 1:1000), MMP-2 (sc-53630; 1:1000), MMP-9 (sc-6840; 1:1000), uPA (sc-14019; 1:1000), cyclin D1 (sc717; 1:1000), and β-actin (sc-47778; 1:2000) were purchased from Santa Cruz Biotechnology (Dallas, Texas). Horseradish peroxidase conjugated anti-mouse and anti-rabbit secondary antibodies were obtained from Promega (Madison, WI). MTT [3-(4,5-dimethylthiazol-2-yl)-2, 5-diphenyltetrazolium bromide] was purchased from Sigma (St. Louis, MO). Recombinant Human TIMP-1 Protein was purchased from R&D Systems, Inc (Minneapolis, MN). Antibody against TIMP-1(#8946; 1:1000) was purchased from Cell Signaling Technology Inc. (Beverly, MA, USA).

### Cell culture

The PC3, DU145, 22Rv1 and LNCap cell lines were purchased from thewere purchase from Bioresources Collection and Research Center, Food Industry Research and Development Institute (Hsinchu, Taiwan). PC3, 22Rv1 and LNCap cells were maintained in RPMI 1640 mediums and DU145 cells were maintained in MEM mediumswith 10% FBS and containing 100 U/ml penicillin, 10 mM HEPES, 0.1 mM NEAA and 1 mM sodium pyruvate, The cultures were incubated at 37°C in a humidified atmosphere of 5% CO_2_. Cells were passaged every 2~3 days to obtain an exponential growth.

### RNA isolation and qRT-PCR analysis

Total cell RNA was extracted by Trizol reagent (Invitrogen, Canada), and their concentrations were determined by microplate spectrophotometer. Reverse transcription was performed following protocol of Applied Biosystems. Quantitative PCR (qPCR) was performed using the Platinum^®^ Quantitative PCR System (Invitrogen) with an ABI 7500 system (Applied Biosystems, Foster City, CA, USA). The following primers were used, ESM1:5′-TT GCTACCGCACAGTCTCAG′ (sense) and 5′-AGGGGA ATTTCAGGCATTTT-3′ (antisense). The expression levels of target genes were normalized to GAPDH gene. The relative quantification of gene expression was determined by triplicate reactions in two independent experiments.

### Gene knockdown by short hairpin RNA (shRNA)

The shRNA delivered by lentivirus system from National RNAi Core Facility (Academia Sinica, Taiwan) was used to knock down ESM1 gene according to the manual. Briefly, to generate the lentivirus containing specific shRNA, HEK-293T cells were co-transfected with 2.25 μg of pCMV-Δ8.9 plasmid with Gag and Pol genes, 0.25 μg of pMD.G plasmid with VSV-G gene, and 2.5 μg of pLKO.1-ESM1 plasmid with specific shRNA for 24 h. The cultured medium containing lentivirus was collected and stored at –80°C as aliquots for further use. To deliver the specific shRNA construct, PC3 and DU145 cells were infected with the lentivirus bearing specific shRNA in culture medium containing 8 μg/mL polybrene and incubated at 37°C for 24 h, then selected in a medium containing 2 or 10 μg/mL of puromycin. Surviving cells were pooled and expanded for analyses of ESM1 expression levels.

### MTT assay

The stable shLuc-shESM1-PC3 and shESM1-DU145 cells were seeded at a density of 4 × 10^4^ cells/well in a 24-well plate and cultured for 24 and 48 h. The the medium was replaced with fresh cell culture medium containing 5 mg/mL MTT for 4 h. The formazan crystals were dissolved in DMSO (100 μl/well). The absorbance at 570 nm of each sample was measured by a Multiskan MS ELSA reader (Labsystems, Helsinki, Finland). The cell growth rates were calculated according to the absorbance values.

### Colony formation assay

The stable shLuc-shESM1-PC3 and shESM1-DU145 cells were seeded into 6-well plates for 2 weeks. Colonies composing more than 50 cells were stained with 0.5% crystal violet for 30 min at room temperature. Triplicate independent experiments were performed.

### Migration and invasion assay

Migration assay was performed using a 48-well Boyden chamber (Neuro Probe, Gaithersburg, MD) plated with 8.0 um pore size polycarbonate membrane filters (Neuro Probe). The lower compartment was filled with RPMI medium containing 20% FBS. The stable shLuc-shESM1-PC3 and shESM1-DU145 cells (2 × 10^4^ cells/ well) were placed in the upper part of the Boyden chamber and incubated at 37°C for 24 h. After incubation, the cells were fixed with methanol for 30 min and stained with 0.05% Giemsa for 1 h; For Invasion assay the upper side was pre-coated with 10 μg/mL Matrigel (BD Biosciences, Bedford, MA). The experimental procedures were the same as in the migration assay.

### Immunofluorescence staining

The shLuc or shESM1 cells were seeded on the an 8 well Lab-Tek Chambered coverglass (Thermo, Rochester,NY) and fixed with 4% paraformaldehyde in PBS for 15 min, permeabilized with 0.1% Triton X-100 for 15 min, and blocked with 1% BSA for 1 h. Cells were stained for indicated primary antibodies and secondary antibody. Subsequently, the slides were mounted in the mounting solution (Invitrogen) containing DAPI for counterstaining cell nucleus and then visualized using an immunofluorescence microscopy.

### Western blot analysis

Cell lysates were extracted in lysis buffer and quantified using the Bradford method, then fractionated by SDS-PAGE. The proteins were transferred to PVDF membranes, incubated with primary antibody and then incubated with a horse radish peroxidase (HRP)-conjugated anti-mouse, anti-rabbit and anti-goat secondary antibody. Protein bands were visualized using ECL chemiluminescent reagent (Millipore, Billerica, USA) and detected using a Luminescent Image Analyzer LAS-4000 mini.

### Tumorigenicity assay and metastasis assay in nude mice

Male BALB/c nude mice (5-weeks old, five mice per group), were purchased from the National Laboratory Animal Center (Taipei, Taiwan). All animal studies were conducted according to the protocols approved by the Institutional Animal Care and Use Committee (IACUC) of Chung Shan Medical University (IACUC Approval No. 1451). The stable shLuc-shESM1-PC3 or shESM1-DU145 cells (5 × 10 ^6^/100 μl) were inoculated into the right flank of mice. Tumor size was measured by every 7 days using a digital vernier caliper. Tumor volume was calculated with the formula 1/2 L1(L2)^2^. Then the mice were sacrificed after 6 weeks. For the *in vivo* metastasis assay, approximately 1 × 10^6^ cells were injected via the tail vein. After 10 weeks, all mice were sacrificed. The lungs were fixed in 4% paraformaldehyde and stained with hematoxylin and eosin (H&E). Lung metastasis was counted and quantified in random selection of high-power fields.

### Statistical analysis

Data are expressed as mean ± SEM. Differences between means were compared by one way ANOVA. *p <* 0.05 was considered statistically significant for all analyses.

## SUPPLEMENTARY MATERIALS FIGURES AND TABLES


