# Supplementary material for: Loss of endothelial cell-specific molecule 1 promotes the tumorigenicity and metastasis of prostate cancer cells through regulation of the TIMP-1/MMP-9 expression
Source: Oncotarget. 2017 Jan 17;8(8):13886–97. doi: 10.18632/oncotarget.14684 (PMC5355147; doi:10.18632/oncotarget.14684)
Supplement: Supplementary file 1 [file oncotarget-08-13886-s001.pdf]

Loss of endothelial cell-specific molecule 1 promotes the tumorigenicity and metastasis of prostate cancer cells through regulation of the TIMP-1/MMP-9 expression

Supplementary Materials

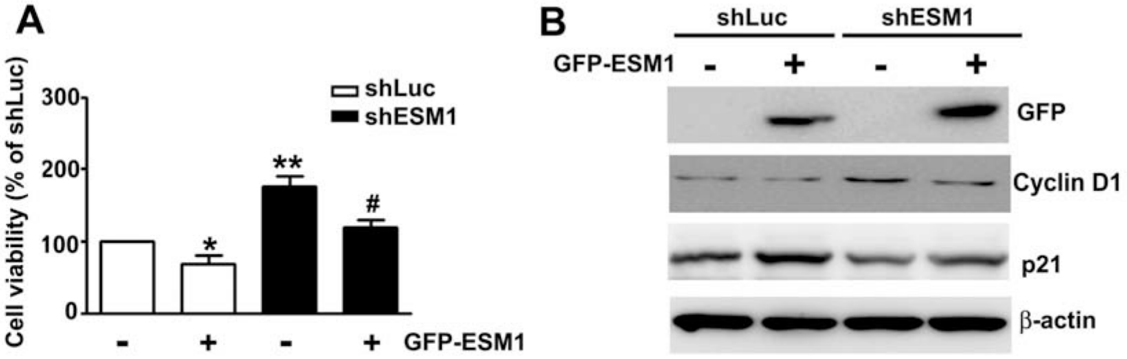

Supplementary Figure 1: (**A**) MTT assay results show that the cell proliferation ability in shESM1-DU145 cells was decreased by GFP-ESM1 transfection. (**B**) The expressions of GFP-ESM1, cyclin D1 and p21 proteins were determined by immunoblotting. Data were presented as the mean ± SE of at least three independent experiments. *$p < 0.05$, **$p < 0.01$, compared with shLuc cells. [#]$p < 0.01$, compared with shESM1 cells.

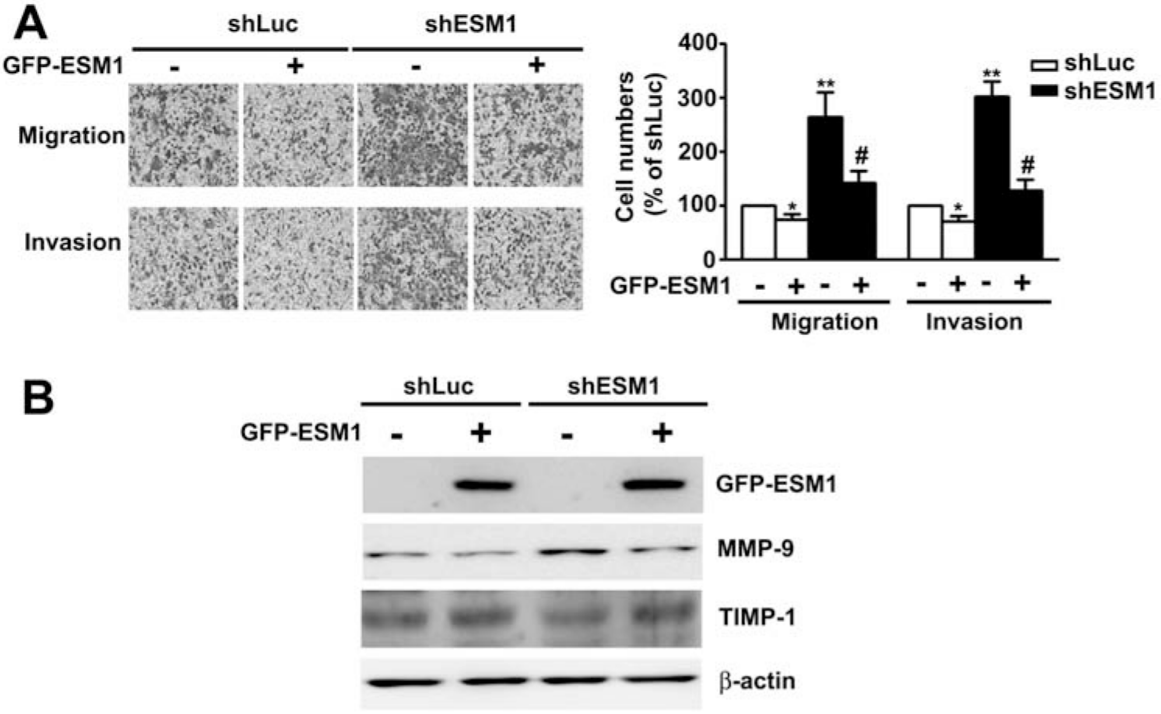

Supplementary Figure 2: ESM1 regulated cells migration/invasion and MMP-9/ TIMP-1 in prostate cancer cells. (**A**) Cell migration and invasion were determined by *in vitro* migration and invasion assay. (**B**) Cell lysates were collected and western blots used to test GFP-ESM1, MMP-9 and TIMP-1 expression in shESM1-DU145 cells. Data were presented as the mean ± SE of at least three independent experiments. *$p < 0.05$, **$p < 0.01$, compared with shLuc cells. [#]$p < 0.01$, compared with shESM1 cells.
